# Editorial: Drug Re-purposing for the Treatment of Bacterial and Viral Infections

**DOI:** 10.3389/fcimb.2019.00387

**Published:** 2019-11-13

**Authors:** Rodolfo García-Contreras, Thomas K. Wood, Maria Tomás

**Affiliations:** ^1^Department of Microbiology and Parasitology, Faculty of Medicine, National Autonomous University of Mexico, Mexico City, Mexico; ^2^Department of Chemical Engineering, Pennsylvania State University, University Park, PA, United States; ^3^Department of Microbiology, Instituto de Investigación Biomédica, Complejo Universitario a Coruña, Universidad de a Coruña, A Coruña, Spain

**Keywords:** antimicrobial peptides, phages, anti-tumoral, anti-virulence, drug libraries, antiprotozoal, anthelmintic

In this Research Topic, about the repurposing for the treatment of bacterial and viral infections, 13 works were published on the use of peptides; on channel-inhibitors and both PBox and LOPAC 1280 molecule libraries; on drugs using infectology, oncology, central nervous system, and metabolism, as well as on phage therapy and natural compounds.

In reference to the use of peptides, Baker et al. performed screening using synthetic peptidomimetics in combination with the antibiotics rifampicin and azithromycin (mainly active for Gram positive bacteria) against multidrug resistant *Escherichia coli* and *Klebsiella pneumoniae* in order to identify those combinations that could enhance the antibiotic's activity, finding that two subclasses of α-peptide/β-peptoid hybrids were suitable. Moreover, some of the combinations also potentiated ticarcillin/clavulanate and erythromycin against *E. coli*, and clindamycin against *K. pneumoniae* and increased the sensitivity of *Pseudomonas aeruginosa* ATCC 27853 as well (Baker et al.).

In addition, two drugs were analyzed with antimicrobial agents in this Research Topic from the infectiology field. The first one, pentamidine, which is an antiprotozooal and antifungal compound, was analyzed alone and in combination with antimicrobials (aminoglycosides, rifampicin, tigecycline, and doripenem) in strains of carbapenemase-producing and/or colistin-resistant *Enterobacteriaceae* (*Klebsiella pneumoniae, Escherichia coli*, and *Enterobacter cloacae*) (Cebrero-Cangueiro et al.). Pentamidine plus rifampicin was the combination that showed synergism in the strains analyzed, making it a new alternative for the treatment of infections caused by carbapenemase-producing and/or colistin-resistant *Enterobacteriaceae*. For the second drug, niclosamide, an anthelmintic drug increased the effects of colistin against colistin sensitive and resistant strains of *A. baumannii* and *K. pneumoniae*, the authors showed that this potentiation is due an increase in the negative bacterial surface charge which favors electrostatic interactions with cationic colistin (Ayerbe-Algaba et al.).

Compounds from two libraries of the molecules were also studied in this Research Topic, the PBox library and LOPAC 1280 library. The Open Access Pathogen Box (PBox) presents a platform to identify new treatments against antibiotic-resistant bacteria through repurposing (Bhandari et al.). Bhandari et al. analyzed the PBox library, which is composed of ~400 compounds, finding 13 compounds with potent antibacterial activity against planktonic and biofilm of isolates of *S. aureus*, ATCC 29213 (methicillin sensitive) and ATCC 700699 (methicillin resistant). These compounds were not cytotoxic for the mouse macrophage cell line, RAW264.7. Of the 13 compounds, two of them (MMV687251 and MMV676477) have structural similarity to vancomycin when comparing their fingerprints of atomic pairs using the Tanimoto coefficient method (Bhandari et al.). Using the LOPAC 1280 and NPC libraries, Cheng et al. found that seven approved drugs with non-antimicrobial indications were effective against *Acinetobacter baumannii* strain AB5075, a highly virulent and MDR strain (resistant to 25 first-line antibiotics for Gram-negatives). Moreover, of the seven candidates, three of them (5-fluorouracil, fluspirilene, and Bay 11-7082) restored the sensitivity of the strain against azithromycin and colistin (Cheng et al.).

For phage therapy, three reviews have been published in this Research Topic. In the first one, Fernández et al. analyzed the current applications of phages and phage-derived lytic proteins in veterinary medicine (prevention and treatment of animal infectious diseases), agriculture (control of bacterial plant diseases), food safety (control of zoonotic bacteria in primary production, sanitizing, and disinfection as well as food biopreservation), and finally, environmental protection (wastewater treatment). In this work, several products from phage therapy were analyzed against pathogens such as *Salmonella, Clostridium perfringens, Escherichia coli* O157:H7, and *Listeria monocytogenes* (Fernández et al.). In the second review, Furfaro et al. studied the clinical trials and regulatory hurdles for bacteriophages therapy (including lysins and magistral phages) for *A. baumannii, P. aeruginosa* and *Enterobacteriaceae*. Moreover, the authors provided a current summary of human phage therapy trials and the range of target sites/infections obtained in the www.clinicaltrials.gov website: Chronic otitis, diarrhea and gastrointestinal disorders, diabetic foot and venous leg ulcers, infected burn wounds, life-threatening as well as urinary tract infections (Furfaro et al.). In the last work, Tagliaferri et al. performed a deep overview about studies using phage-antibiotic combinations (ß-lactams, aminoglycosides, fluorquinolones, polymyxins, tetracyclines, and others) against pathogenic bacteria separated *in vitro* (plaque, planktonic, and biofilm) and *in vivo* (human, mouse/rat, broiler, and *Galleria mellonella*) as well as studies for Gram negative strains such as *P. aeruginosa, Escherichia coli, Klebsiella pneumoniae, Burkholderia cepacia*, and Gram positive isolates, such as *Staphylococcus aureus* and *Enterococcus faecalis* (Tagliaferri et al.).

In a theoretical work, Mujawar et al. explored the potential of 19 anti-typhoid- drugs, two phenalenone-furanone derivatives and other 15 drugs with phenalenone or furanone moieties mostly used against Gram positive bacteria to bind and inhibit a set of seven important chaperones or members of two component signal transduction systems (including DnaK, SicA, and EnvZ), finding that XR770, one of the two phenaleno-furanone moiety-based derivatives included, had the best binding interactions and stability suggesting it is the best candidate for a new anti-*Salmonella* drug. Nevertheless, experimental work is needed in order to validate this finding.

For combat-related infections, to prevent them is pivotal, and in this regard, Liu et al. focused on the design of a novel antimicrobial coating for intravascular catheters, which are the main source of nosocomial bacteremia and caused mainly by Gram positive bacteria. Interestingly, the active component of this coating is the antirheumatic drug auranofin which also has antibacterial antifungal and anti-biofilm activities due the inhibition of thioredoxin reductase causing a Redox imbalance. This novel coating that also includes polyurethane as a drug carrier was able to inhibit the growth of MRSA and its biofilm formation *in vitro*, and was also biocompatible with both erythrocyte and liver cells (Liu et al.).

Gallium nitrate is commonly used to treat hypercalcemia malignancy, and previously it was shown that it had remarkable antibacterial properties against *P. aeruginosa* and *A. baumannii*, interfering with iron transport and metabolism, hence making it a suitable drug for repurposing. In their work, Hijazi et al. performed the first comparison of the effects of gallium nitrate and another two gallium formulations gallium maltolate and Ga(III)-protoporphyrin IX (GaPPIX) in strains of all the ESKAPE bacteria in different culture media including RPMI supplemented with human serum with low free iron concentrations and which mimic the *in vivo* environment. Interestingly, gallium maltolate and gallium nitrate had similar activities in this medium but GaPPIX lost its activity due its complexation with albumin. Moreover, this work allowed the identification of the *hemO* gene cluster as the main route for the intake of GaPPIX in *A. baumanii* (Hijazi et al.).

According to the discovery of suitable drug candidates for its repurposing as antibacterial compounds, Radosevic et al. performed a virtual sequential screening of 2,627 approved small compounds as inhibitors of the M2 channel protein of the Influenza A virus that were able to inhibit the wild-type channel as well as a channel with the S31N mutation that rendered resistant to adamantanes (the canonical blockers of the M2 channel), finding five potential candidates. One of the best candidates, guanethidine, successfully decreased viral titers of H1N1 infected cells (Radosevic et al.).

Finally, in this Research Topic, a review was presented about drug repurposing from infectology (antiretroviral, anthelminthic, antiprotozoal, and antifungal), oncology (antineoplastic and selective estrogen receptor modulator), the central nervous system (antidepressant and antipsychotic), metabolism (anti-inflammatory and diuretic), and natural compounds (stilbene, anticancer, antioxidant, and essential oil) to fight colistin and carbapenem-resistant belonging ESKAPE-group bacteria (*Enteroccocus faecium, S. aureus, K. pneumoniae, A. baumannii, P. aeruginosa, Enterobacter* species) (Peyclit et al.).

In conclusion, “re-purposing” (defined as investigating new uses for existing approved drugs) has gained renewed interest, as reflected in the works published in this this special issue of Frontiers in Cellular and Infection Microbiology which may help to speed up the drug development process and save years of expensive research invested in antimicrobial drug development.

## Author Contributions

RG-C and MT wrote the manuscript using papers revised as editors in this Research Topic. TW participated in the supervision of the writing of the manuscript.

### Conflict of Interest

The authors declare that the research was conducted in the absence of any commercial or financial relationships that could be construed as a potential conflict of interest.

